# Optimization of Corn Resistant Starch Preparation by Dual Enzymatic Modification Using Response Surface Methodology and Its Physicochemical Characterization

**DOI:** 10.3390/foods11152223

**Published:** 2022-07-26

**Authors:** Yangjin Liu, Fan Jiang, Chunwei Du, Mengqing Li, Zhifu Leng, Xiuzhu Yu, Shuang-Kui Du

**Affiliations:** Engineering Research Center of Grain and Oil Functionalized Processing in Universities of Shaanxi Province, College of Food Science and Engineering, Northwest A&F University, 22 Xinong Road, Xianyang 712100, China; liuyangjin321@163.com (Y.L.); jiangfan9592@163.com (F.J.); duchunwei08@163.com (C.D.); limengq@nwafu.edu.cn (M.L.); lzf891023@163.com (Z.L.); xiuzhuyu@nwafu.edu.cn (X.Y.)

**Keywords:** resistant starch, thermostable α-amylase, pullulanase, enzymatic modification, response surface, physicochemical properties

## Abstract

Corn starch was dually modified using thermostable α-amylase and pullulanase to prepare resistant starch (RS). The concentration of starch liquid, the amount of added thermostable α-amylase, the duration of enzymatic hydrolysis and the amount of added pullulanase were optimized using RSM to increase RS content of the treated sample. The optimum pretreatment conditions were 15% starch liquid, 3 U/g thermostable α-amylase, 35 min of enzymatic hydrolysis and 8 U/g pullulanase. The maximum RS content of 10.75% was obtained, and this value was significantly higher than that of native corn starch. The degree of polymerization (DP) of the enzyme-modified starch decreased compared with that of native starch. The scanning electron microscopy (SEM) and differential scanning calorimetry (DSC) were performed to assess structural changes in native and pretreated starch. The effect of dual enzyme pretreatment on the structure and properties of corn starch was significant. Unlike the untreated one, the pretreated corn starch showed clear pores and cracks. Significant differences in RS contents and structural characterization between starch pretreated and untreated with dual enzymes demonstrated that the dual enzyme modification of corn was effective in enhancing RS contents.

## 1. Introduction

Starch is a vital carbohydrate for human health in nature. Based on its nutritional properties and release rate of glucose, starch can be classified into rapidly digestible starch, slowly digestible starch and resistant starch (RS) [1]. Among these categories, RS has recently gained much attention in the food industry due to its ability to enhance satiety, stabilize blood glucose and its important role in combating metabolic diseases and obesity. RS cannot be digested in the small intestine, but it can react with volatile fatty acids in the gastrointestinal tract, causing a decrease in human intestinal pH, thus helping to protect the mucosa and effectively promoting intestinal motility [2,3]. RS can be divided into five categories (RS1-RS5) based on the physicochemical and structural characteristics of starch granules, granularity, cooking operations and interactions with other components. RS1 is inaccessible starch entrapped within whole or partly milled grains or seeds and tubers among all types of RS [4]. RS2 is native starch with a B- or C-polymorph granule whose special structure makes them hydrolyze under stringent conditions. RS3 comprises retrograded starch formed during heating and aging. RS4 refers to the starches chemically modified or repolymerized, including conversion, substitution or cross-linking, which resist enzymatic hydrolysis because starch contains specific chemical linkages. RS5 is formed by the interaction of amylose starch with lipids, fats and surface-active agents [5,6]. Physical, chemical and enzymatic methods of producing RS have been investigated [7]. Among these methods, enzyme modification techniques have the advantages that the reaction conditions are mild and controlled and do not require high-pressure and acid-resistant equipment, making it safe for the environment and consumers. Moreover, they have many typical reactions with few types of by-products formed by enzymatic processing. Sun et al. [8] used three treatments (autoclaving, pullulanase and pullulanase-autoclaving treatments) to prepare RS from purple sweet potatoes. This research offered a theoretical foundation for developing purple sweet potatoes’ RS preparation. Reddy et al. [9] prepared RS by the enzymatic method using red kidney beans (*Phaseolus vulgaris. L*) as raw material. According to their findings, enzymatic digestion and heat treatment of starch boosted the production of RS. Das et al. [10] focused on increasing the intrinsic content of RS in green banana flour using a mixed enzyme treatment. The molecular conversion of starch grains was effectively increased by utilizing amylase and amyloglucosidase. Furthermore, Simons et al. [11] used beans and corn to produce retrograded RS3, and they found no significant differences between the α-amylase and pullulanase treatments.

Thermostable α-amylase cleaves α-1,4 glucans into linear chains that can rapidly decrease the viscosity of gelatinous starch solution and promote the production of soluble dextrin and oligosaccharides [12]. Excessive hydrolysis by α-amylase can produce a small amount of glucose and maltose. Pullulanase (pullulan 6-glucanohydrolase) is a debranching enzyme used in starch modification processes. It can specifically cleave α-1,6 glycosidic linkages in pullulan, amylopectin and numerous related polysaccharides [13,14]. Pullulanase has been used to produce high amylose starches, which have considerable commercial value [15]. The increase in the starch branch density and the crystalline structure of starch by enzymatic modification both contribute to delayed digestion [16]. Dually modified starch had higher RS contents than native corn starch [17]. Corn is one of the most vital starch-producing crops worldwide. Corn starch mainly comprises amylopectin that can be converted into short linear glucans during pullulanase treatment, thereby promoting the production of RS [18]. The dual enzyme modification of starch can be used to increase the content of corn RS and promote the development of novel food industry and dietary customs by considering their anti-digestion properties [19].

Response surface methodology (RSM) is a statistical method for solving multivariate factors. Specific data are obtained through the test, and a polynomial quadratic equation is used to fit a functional relationship between the variables and response. The fitted polynomial equation is analyzed to find the optimum process parameters. Wang et al. [20] developed and optimized a new method for ultrasound-assisted enzyme extraction of kiwi starch using RSM. Kaur et al. [21] prepared RS from green banana peels with the ultrasound-assisted enzymatic extraction. The effects of process parameters such as treatment time, temperature and liquid-to-solid ratio on the yield of RS were investigated using RSM and the Box–Behnken design. Kahraman et al. [22] studied the optimization of the best reaction condition for preparing high RS content from cross-linked starch by using RSM. RSM allows not only to explore the optimal process, but also to further analyze in depth the extent to which different processes affect the starch properties. However, there are few reports on the application of RSM to analyze the enzymatic extraction of resistant starch from corn, and this direction is valuable for research.

In this study, corn starch subjected to dual enzyme modification by using thermostable α-amylase and pullulanase was investigated using response surface methodology (RSM). Factors affecting enzymatic modifications were optimized by a single-factor test and RSM. Structural characteristics were assessed by scanning electron microscopy (SEM), differential scanning calorimetry (DSC) and degree of polymerization (DP). This study aimed to optimize the dual enzyme modification conditions of corn starch to increase the content of RS and to reveal the structural differences between native and modified corn starch. 

## 2. Materials and Methods

### 2.1. Materials

The native corn starch with 27.9% amylose was obtained from a large supermarket (Xi’an Guowei Starch Company, Xi’an, China). Its moisture content and protein content were 10.7% and ≤0.2%, respectively. Thermostable α-amylase (1400 U/mg) from porcine pancreas (type VI-B), pullulanase (1000 U/mL) and dinitrosalicylic acid (DNS) were purchased from Sigma–Aldrich Chemical Co. (St. Louis, MO, USA). A Megazyme Resistant Starch Assay Kit was purchased from Megazyme Int. Ireland Ltd., Co. (Wicklow, Ireland). All other reagents were of analytical grade.

### 2.2. Methods

#### 2.2.1. Preparation of RS 

Corn RS was prepared in accordance with the methods of Zhang et al. [23] with some modifications. Starch liquid of different concentrations made from corn starch and phosphate buffer (pH 6.4) was gelatinized by heating in an autoclave sterilizer (ES-315, Tomy Kogyo Co., Ltd., Tokyo, Japan) at 121 °C. The thermostable α-amylase was added to the starch liquid at 90 °C for various duration. Then, the mixture was treated with pullulanase and incubated in a shaking water bath at 60 °C for 12 h. The starch liquid was then cooled to room temperature and stored at 4 °C for 18 h. The obtained starch was dried at 60 °C for 24 h, ground and passed through an 80-mesh sieve for further analysis.

#### 2.2.2. Determination of RS

The RS content was measured using the Megazyme Resistant Starch Assay Kit based on the AACC-approved method 32-40 [24]. Samples were incubated with α-amylase and amyloglucosidase (AMG) for 16 h at 37 °C to solubilize and hydrolyze non-RS to glucose. RS was recovered through centrifugation (3800 r/min, 20 min) and then dissolved in KOH and hydrolyzed to glucose with amyloglucosidase. The absorbance of glucose was then determined with a glucose oxidase reagent at 510 nm. RS was calculated as glucose × 0.9.

#### 2.2.3. Single-Factor Experiment 

In order to determine the effects of different conditions on RS content, the following factors were carried out at various starch liquid concentrations (5, 10, 15, 20 and 25%), the amount of added thermostable α-amylase (0, 2, 4, 6 and 8 U/g), the duration of enzymatic (thermostable α-amylase) hydrolysis (15, 25, 35, 45 and 55 min) and the amount of added pullulanase (4, 6, 8, 10 and 12 U/g). Then, followed the step in Section 2.2.1 and Section 2.2.2 to prepare RS and determined the RS content. The maximum RS content in the final extract was used as the best condition to design subsequent experiments.

#### 2.2.4. Experimental Design and Statistical Analysis

RSM is a collection of mathematical and statistical technique used to model and analyze problems in which a response of interest is influenced by several variables, and the objective of this technique is to optimize this response [25]. Significant factors, including the concentration of starch liquid, the amount of added thermostable α-amylase, duration of enzymatic (thermostable α-amylase) hydrolysis and amount of added pullulanase, were selected based on the results of the single-factor test as major variables for further analysis. A polynomial quadratic equation was used to evaluate the mathematical relationship between the variables and response.

Y = b_0_ + b_1_X_1_ + b_2_X_2_+b_3_X_3_ +b_4_X_4_+ b_11_X_1_^2^ + b_22_X_2_^2^ + b_33_X_3_^2^ + b_44_X_4_^2^+ b_12_X_1_X_2_ +b_13_X_1_X_3_ +b_14_X_1_X_4_ + b_23_X_2_X_3_+ b_24_X_2_X_4_+ b_34_X_3_X_4_ (1)

where Y is the RS content; X_1_, X_2_, X_3_ and X_4_ are the concentration of starch liquid, the amount of added thermostable α-amylase, duration of enzymatic (thermostable α-amylase) hydrolysis and amount of added pullulanase, respectively; b_0_ is the interception coefficient; b_1_, b_2_, b_3_ and b_4_ are linear coefficients; b_12_, b_13_, b_14_, b_23_, b_24_ and b_34_ are interaction coefficients; and b_11_, b_22_, b_33_ and b_44_ are the quadratic coefficients. These coefficients represent the linear, quadratic and interaction effects of the variables, respectively. The fitted polynomial equation was calculated to visualize the relationship between the response and experimental levels of each factor and to deduce the optimum conditions [26]. Analysis of variance (ANOVA) tables were generated, and the effect and regression coefficients of individual linear, quadratic and interaction terms were determined. The regression coefficients were then used for statistical calculation.

#### 2.2.5. Detection of the Average Degree of Polymerization (DP) of Starch

The average degree of polymerization (DP) of starch samples (native corn starch; enzymatic starch: starch liquid concentration was 15%, duration of enzymatic hydrolysis was 25 min, the amount of added pullulanase was 8 U/g, the amount of added thermostable α-amylase was 2, 4, 6 and 8 U/g) was determined in accordance with the methods of Hizukuri et al. [27] with appropriate modifications. The dry powders of starch samples (0.5 g) were blended with 25 mL of 2 M KOH and shaken up at 25 °C. After a uniform mixture was formed, hydrochloric acid solution (1.00 mol/L) was added until pH was 7.0 and the volume was increased to 50 mL with distilled water. Approximately 1 mL of solution was used to measure the amount of the reducing end by the DNS method. The DP value was determined as the ratio of total glucose and reducing residue concentrations of the starches. The DP of starch was calculated based on the following equation:

(2) $$\mathrm{DP}=\frac{1.1\times W}{50\times G}$$

where G is the content of glucose, w is the weight of starch samples and 1.1 is the conversion value of starch to glucose. Experiments were conducted in duplicate.

#### 2.2.6. Observation of Particle Morphology

The morphologies of native and enzymatic-modified corn starch were observed using an SEM (S-3400N, Hitachi, Japan) at an accelerating voltage of 5.0 kV. Samples were fixed on an aluminum column and held in place with double-sided adhesive carbon tape before sputtering gold to a thickness of 50 nm.

#### 2.2.7. Determination of Thermal Characteristics

The thermal properties of starch samples were determined using a differential scanning calorimeter (DSC; DSC 7000X, Hitachi, Japan) based on the method of Gong et al. [28] with some modifications. First, the instrumental apparatus was calibrated using indium. Then, 3 mg of the samples and distilled water were added to an aluminum pan in a 1:3 mass ratio and sealed, kept for 12 h at 25 °C. Next, the equilibrated aluminum pan was heated from 30 to 150 °C at 10 °C/min in a continuous flow of dry N_2_ gas with a rate of 30 mL/min using an empty aluminum pan as a reference. The transition temperatures of gelatinization (onset, T_0_; peak, T_p_; conclusion, T_c_) and the enthalpy of gelatinization (ΔH) were analyzed using relevant software to characterize the thermal properties of starch samples. 

### 2.3. Statistical Analysis 

All the experiments were conducted in duplicate; the experimental data in this study were expressed as mean ± standard deviation. Data were analyzed with SPSS Statistics 20.0 (IBM, Armonk, NY, USA) and Design Expert 8.06. Analysis of variance (ANOVA) and Least Significant Difference (LSD) were used to determine significant differences (*p* < 0.05).

## 3. Results

### 3.1. Effect of Modification Parameters

#### 3.1.1. Effect of Starch Liquid Concentration

**Figure 1:**
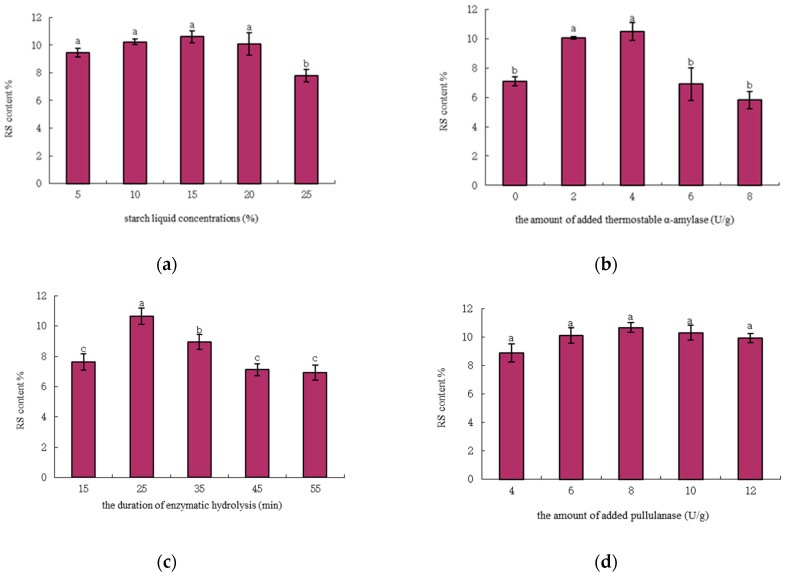
Effect of starch liquid concentrations (**a**), the amount of added thermostable α-amylase (**b**), the duration of enzymatic (thermostable α-amylase) hydrolysis (**c**) and the amount of added pullulanase (**d**) on RS content. Values with different letters in the same graph are significantly different (*p* < 0.05).

The effect of the starch liquid concentration on the RS content is listed in Figure 1a. The content of corn RS initially increased and then decreased as the starch liquid concentrations increased. The RS content increased as the starch liquid concentration increased within the range of 5–15% and decreased significantly above 25%. This finding indicated that the low starch liquid concentration favored enzymatic hydrolysis. At low starch liquid concentrations, the magnitude of the association of thermostable α-amylase and pullulanase with starch molecules became significant. At too high concentrations, thermostable α-amylase and pullulanase cannot react evenly with the starch molecules. Moreover, it will lead to difficulties in starch gelatinization, which is not conducive to the mutual proximity and crystallization of amylose, leading to a reduction in the RS content. The trend of our results was similar to that found by Liu et al. [29] which showed that the content of corn RS did not change significantly with the starch liquid from 10% to 20%. In conclusion, the starch liquid concentration of 15% was beneficial to the enzymatic hydrolysis of starch.

#### 3.1.2. Effect of the Amount of Added Thermostable α-Amylase

In Figure 1b, the content of corn RS initially increased and then decreased as the amount of the added thermostable α-amylase (4–8 U/g) increased. The maximum content of RS was obtained when the amount of added thermostable α-amylase was 4 U/g; the trend of our results was similar to that found by Zhang and Jin [30]. The addition of thermostable α-amylase and pullulanase increased the corn RS content compared to native corn starch without enzymatic hydrolysis (Table 1). However, the excessive increase in the amount of added thermostable α-amylase resulted in the DP of the starch decreasing. Doblado-Maldonado et al. [31] suggested that differences in the fine molecular structure of starch can drive the amount of RS, and starch with higher DP and shorter external chains had a higher content of RS. The high amount of added α-amylase led to a significant decrease in the DP of starch caused by high branching, thereby leading to the reduction in the RS content. This result suggested that thermostable α-amylase should be added properly to increase the RS content effectively.

#### 3.1.3. Effect of the duration of Enzymatic (Thermostable α-Amylase) Hydrolysis

The effect of the duration of enzymatic (thermostable α-amylase) hydrolysis on the RS content is shown in Figure 1c. The effect of the duration of enzymatic (thermostable α-amylase) hydrolysis on the RS content is shown in Figure 1c. The RS content increased significantly as the reaction time increased from 15 min to 25 min, and the maximum was reached at 25 min. However, a marked reduction was observed when the reaction time increased from 25 min to 55 min. Zhang and Jin [30] reported that the initial rate of the dual enzyme reaction increased rapidly. Furthermore, the rate of hydrolysis and RS content decreased with the duration of the enzyme modification being longer than 25 min. The trend of our results was similar to that of Zhang and Jin [30]. Reaction time is one of the essential factors in the enzymatic production of RS. Given sufficient time, amylose molecules could be degraded entirely into glucoside macromolecules, thereby preventing RS formation. Miao et al. [32] indicated that the RS content initially increases because the dual enzyme removes maltose to increase the enzymatic reaction speed and further creates α-1,6 glycosidic linkages. The subsequent decrease in RS content might be caused by the enzyme inhibition of the hydrolysis product. Native corn starch subjected to double enzyme treatment produced more branch structures by adding more α-1,6 linkages on long chains, thereby decreasing RS content. Reddy et al. [9] showed that the RS content increases continuously as the duration of hydrolysis is extended from 10 min to 40 min. The disagreement with our results might be due to the differences in the type of starch or enzyme hydrolysis conditions and the narrow range of hydrolysis time.

#### 3.1.4. Effect of the Amount of Added Pullulanase

The effect of the amount of added pullulanase on the RS content is shown in Figure 1d. When the amount of added pullulanase increased from 4 U/g to 12 U/g, the content of corn RS initially increased and then dropped. This result was consistent with that of Shi et al. [33]. The maximum content was 10.75% when the added amount was 8 U/g (Figure 1d). Ren et al. [34] indicated that pullulanase specifically catalyzes the hydrolysis of α-1,6-glycosidic bonds in amylopectin of native starch, resulting in the disruption of double helices and the loss of the characteristic A-type crystal structure, and the more the amount of added pullulanase, the greater the interaction of the enzyme with the starch granules in the studied range. The increase in the amount of amylose led to an increase in the content of RS. Shi et al. [15] found that the RS content increased sharply as the amount of the enzyme increased. The highest RS content was obtained with 20 U/g pullulanase, but this content decreased at 30 U/g pullulanase. This disagreement with our results might be due to the differences in enzyme hydrolysis conditions or solvents and the narrow range of enzyme concentrations. Overall, 8 U/g pullulanase was used for further analysis.

### 3.2. Response Surface Analysis

RSM results indicated that the RS content varied depending on the given conditions (Table 2). The content varied from 5.02% to 10.75% in different composite experiments. The maximum content was found under the following experimental conditions: X_1_ = 15%, X_2_ = 3 U/g, X_3_ = 35 min and X_4_ = 8 U/g. The maximum RS content of 10.75% was obtained, and this value was significantly higher than that of native corn starch. The quadratic polynomial model of the RS content (Y) comprised the following variables: the concentration of starch liquid (X_1_), the amount of added thermostable α-amylase (X_2_), the duration of enzymatic hydrolysis (X_3_) and the amount of added pullulanase (X_4_).

Y= −3.74 + 0.2401 X_1_+ 4.6548 X_2_+ 0.2221 X_3_ + 0.4846 X_4_ − 0.0102 X_1_^2^ − 0.8985 X_2_^2^ − 0.0035 X_3_^2^ − 0.0335 X_4_^2^+ 0.0010 X_1_X_2_ + 0.0003 X_1_X_3_ + 0.0022 X_1_X_4_ + 0.0063 X_2_X_3_ + 0.0344 X_2_X_4_ − 0.0001 X_3_X_4_ (3)

The ANOVA results of the developed model and regression coefficients are presented in Table 3. *p*-values were used as a tool to check the significance of each coefficient, which in turn might indicate the pattern of the interactions between the variables. The smaller the *p*-value was, the more significant the corresponding coefficient would be. In the present study, the *p*-value of the regression model was less than 0.01 (Table 3), confirming that the model was found to be adequate for prediction within the range of the experimental variables. The variable with the largest effect on influencing the RS content was the quadratic term duration of enzymatic hydrolysis (X_3_^2^) followed by the quadratic term the amounts of added α-amylase (X_2_^2^), quadratic term concentration of starch liquid(X_1_^2^) and linear term the amounts of added α-amylase (X_2_). However, all other interaction terms were found insignificant (*p* > 0.05). The order of independent variables based on their main effects on increasing the RS content is X_2_ > X_3_ > X_1_ > X_4_, which indicates that the amounts of added α-amylase are the most important variable influencing the RS content, followed by the duration of enzymatic hydrolysis and concentration of starch liquid. 

### 3.3. Morphological Properties

**Figure 2:**
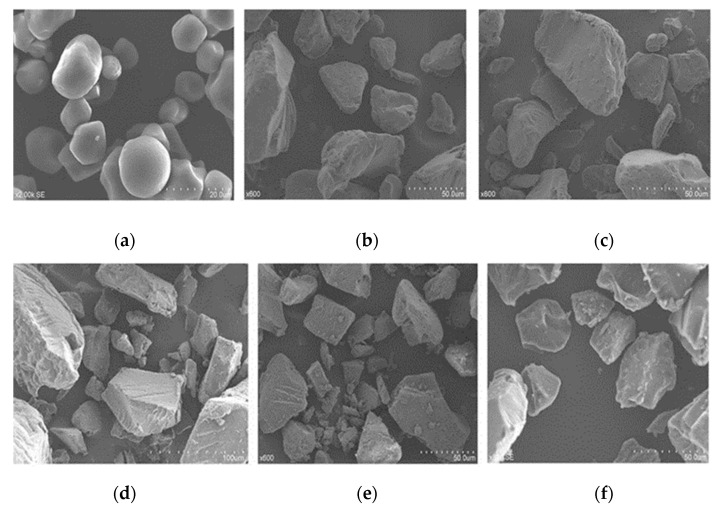
SEM images of (**a**) native corn starch, (**b**) gelatinized corn starch without enzymatic modification (α-amylase, 0 U/g; pullulanase, 0 U/g), (**c**) dual enzyme-treated starch (α-amylase, 2 U/g; pullulanase, 8 U/g), (**d**) dual enzyme-treated starch (α-amylase, 4 U/g; pullulanase, 8 U/g), (**e**) dual enzyme-treated starch (α-amylase, 8 U/g; pullulanase, 8 U/g) and (**f**) single enzyme-treated starch (α-amylase, 0 U/g; pullulanase, 8 U/g).

The surface of the starch granules of RS was investigated through SEM. The surface of native starch granules was displayed as round or oval granular shapes without evidence of cracks (Figure 2a). Some cracks appeared on the surface of the autoclaved starch. (Figure 2b). The surface of the enzymatically-hydrolyzed sample showed significant signs of cracks and a rough surface. More granular degradation occurred in RS as the amount of the added α-amylase increased (Figure 2c–e). Single enzyme-treated samples by pullulanase showed irregular-shaped fragments (Figure 2f). However, the damage degree of single enzyme-treated samples was weaker than that of dual enzyme-treated ones. Enzymatic hydrolysis might favor the breaking of α-1,4 glucans at the center of the starch granules into linear chains and shorten the starch chain. Excessive α-amylase preferentially attacked linear chains of corn starch. Then, these starches formed smaller particles during aging and coagulation [35]. Amylopectin was converted into amylose because pullulanase catalyzed the hydrolysis of α-1,6 glycosidic bonds during RS preparation, and some molecular structures were destroyed [36]. After amylose was dissolved, amylose crystals were formed during the subsequent aging process. 

### 3.4. Thermal Characteristics

The thermal parameters and the enthalpy change (ΔH) are summarized in Table 4. Significant differences (*p* < 0.05) were observed in T_0_, T_p_, T_c_ and ΔH among the samples. Native corn starch showed a specific gelatinization pattern with a large gelatinization endotherm appearing at approximately 60 °C because of the melting of crystallites in native starch granules [37]. The transition onset (T_0_), peak (T_p_) and conclusion temperature (T_c_) and the transition enthalpy (ΔH) of treated samples increased significantly as compared with the native starch. Comparatively, the melting temperature of the dually-modified starches was higher than that of the single pullulanase-treated starch. The increase in ΔH can be explained in terms of increased ordering and stabilization of double helical structures through hydrogen bonds and other intermolecular forces [38]. Hence, the gelatinization temperature of the RS product was increased and showed more thermal stability than native starch. The trend of our results was similar to Doan et al. [39], who found that the enzyme-modified starches displayed higher melting and viscosity peak temperatures than did native starch counterparts. Dual enzyme modification could promote the formation of double helices that contributed to the production of the typical crystalline structure in starches by internal chain association [34].

## 4. Conclusions

The combined enzymatic modification of corn starch with thermostable α-amylase and pullulanase resulted in a marked increase in RS. The amount of added thermostable α-amylase was the most important factor that influenced the RS content, and this factor was followed by the duration of enzymatic hydrolysis, concentration of starch liquid and the amount of added pullulanase. A maximum RS content of 10.75% was obtained using 15% starch liquid, 3 U/g thermostable α-amylase, 35 min of enzymatic hydrolysis and 8 U/g pullulanase. This value was significantly higher than that of native and single enzyme-treated starch. SEM image, DSC and DP determination results demonstrated the remarkable effects of the dual enzyme modification pretreatment of corn RS.

## Figures and Tables

**Table 1:** Effect of different thermostable α-amylase adding amounts on RS content and DP ^#1^.

Amount of Added Thermostable α-Amylase U/g	DP	RS Content %
native corn starch	298 ± 5.56 ^a^	1.14 ± 0.09 ^d^
2	157 ± 7.58 ^b^	10.06 ± 0.10 ^a^
4	90 ± 5.90 ^c^	10.52 ± 0.31 ^a^
6	56 ± 7.07 ^d^	6.91 ± 0.89 ^b^
8	24 ± 4.61 ^e^	5.82 ± 0.48 ^c^

^#1^: starch liquid concentration was 15%, duration of enzymatic hydrolysis was 25 min and the amount of added pullulanase was 8 U/g. Data are the mean ± standard deviation (*n* = 2). Values with different letters in the same column are significantly different (*p* < 0.05).

**Table 2:** RSM for optimization of parameters for preparation of corn starch RS.

No.	Concentration of Starch Liquid (%)X_1_	Amount of Added Thermostable α-Amylase (U/g) X_2_	Duration of ENZYMATIC hydrolysis (min) X_3_	Amount of Added Pullulanase (U/g) X_4_	RS Content %
1	5	2	15	4	7.42
2	5	4	15	4	6.52
3	25	2	15	4	6.06
4	25	4	15	4	5.51
5	5	2	55	4	6.92
6	5	4	55	4	6.67
7	25	2	55	4	5.97
8	25	4	55	4	5.02
9	5	2	15	12	7.77
10	5	4	15	12	7.24
11	25	2	15	12	6.64
12	25	4	15	12	6.07
13	5	2	55	12	6.97
14	5	4	55	12	7.02
15	25	2	55	12	6.07
16	25	4	55	12	6.67
17	15	1	35	8	7.89
18	15	5	35	8	6.71
19	0	3	35	8	8.22
20	35	3	35	8	6.78
21	15	3	75	8	5.53
22	15	3	35	0	7.84
23	15	3	35	16	9.66
24	15	3	35	8	10.62
25	15	3	35	8	10.75
26	15	3	35	8	10.51
27	15	3	35	8	10.63
28	15	3	35	8	10.42
29	15	3	35	8	10.68

**Table 3:** ANOVA for response surface quadratic model.

Source	Sum of Squares	Degree of Freedom	Mean Square	*F*-Value	*p*-Value prob > *F*
Model	86.5004	14	6.1786	21.18	<0.001
*X*_1_	3.6038	1	3.6038	12.36	0.003
*X_2_*	11.0703	1	11.0703	37.95	<0.001
*X*_3_	10.2196	1	10.2196	35.04	<0.001
*X*_4_	2.4574	1	2.4574	8.42	0.012
*X*_1_^2^	19.1035	1	19.1035	65.49	<0.001
*X_2_* ^2^	21.7944	1	21.7944	74.72	<0.001
*X*_3_^2^	31.4824	1	31.4824	107.93	<0.001
*X*_4_^2^	7.7561	1	7.7561	26.59	<0.001
*X*_1_ *X*_2_	0.0016	1	0.0016	0.01	0.942
*X*_1_ *X*_3_	0.0420	1	0.0420	0.14	0.710
*X*_1_ *X*_4_	0.1260	1	0.1260	0.43	0.522
*X*_2_ *X*_3_	0.2500	1	0.2500	0.86	0.370
*X*_2_ *X*_4_	0.3025	1	0.3025	1.04	0.326
*X*_3_ *X*_4_	0.0002	1	0.0002	0.00	0.978
Residual	4.0836	14	0.2917		
Cor Total	90.5840	28			

**Table 4:** Thermal transition properties of native and enzyme-treated starches.

Treatment	RS Content (%)	T_0_ (°C)	T_P_ (°C)	T_C_ (°C)	△H (J/g)
Native corn starch	1.14 ± 0.09 ^d^	57.18 ± 0.17 ^e^	61.55 ± 0.15 ^e^	66.60 ± 0.11 ^e^	5.22 ± 0.09 ^e^
Gelatinized corn starch ^#1^	4.38 ± 0.11 ^c^	63.59 ± 0.11 ^d^	68.36 ± 0.21 ^d^	75.53 ± 0.21 ^d^	6.71 ± 0.10 ^d^
Dual enzyme-treated ^#2^	7.66 ± 0.53 ^b^	102.63 ± 0.21 ^b^	104.43 0.17 ^b^	106.09 0.25 ^b^	14.68 ± 0.08 ^c^
Dual enzyme-treated ^#3^	10.52 ± 0.31 ^a^	135.66 ± 0.24 ^a^	135.76 ± 0.24 ^a^	143.01 ± 0.19 ^a^	19.11 ± 0.11 ^a^
Pullulanase-treated ^#4^	7.18 ± 0.31 ^b^	76.3 ± 0.21 ^c^	91.01 ± 0.32 ^c^	100.57 ± 0.26 ^c^	15.81 ± 0.21 ^b^

^#1^: starch liquid concentration was 15%, the amount of added thermostable α-amylase was 0 U/g, the amount of added pullulanase was 0 U/g; ^#2^: starch liquid concentration was 15%, the amount of added thermostable α-amylase was 4 U/g, the duration of enzymatic hydrolysis was 15 min, the amount of added pullulanase was 8 U/g; ^#3^: starch liquid concentration was 15%, the amount of added thermostable α-amylase was 4 U/g, the duration of enzymatic hydrolysis was 25 min, the amount of added pullulanase was 8 U/g; ^#4^: starch liquid concentration was 15%, the amount of added thermostable α-amylase was 0 U/g, the amount of added pullulanase was 8 U/g; data are the mean ± standard deviation (*n* = 2). Values with different letters in common in the same column are significantly different (*p* < 0.05).

## Data Availability

Data are contained within the article.
